# Which Sugar to Take and How Much to Take? Two Distinct Decisions Mediated by Separate Sensory Channels

**DOI:** 10.3389/fnmol.2022.895395

**Published:** 2022-06-03

**Authors:** Soh Kohatsu, Noriko Tanabe, Daisuke Yamamoto, Kunio Isono

**Affiliations:** ^1^Neuro-ICT Laboratory, Advanced ICT Research Institute, National Institute of Information and Communications Technology, Kobe, Japan; ^2^Fukuoka Junior College for Kindergarten Teachers, Fukuoka, Japan; ^3^Graduate School of Life Sciences, Tohoku University, Sendai, Japan

**Keywords:** *Drosophila*, gustatory receptor neurons, feeding behavior, Gr5a, Gr61a, sweet taste

## Abstract

In *Drosophila melanogaster*, gustatory receptor neurons (GRNs) for sugar taste coexpress various combinations of gustatory receptor (*Gr*) genes and are found in multiple sites in the body. To determine whether diverse sugar GRNs expressing different combinations of *Gr*s have distinct behavioral roles, we examined the effects on feeding behavior of genetic manipulations which promote or suppress functions of GRNs that express either or both of the sugar receptor genes*Gr5a* (*Gr5a*+ GRNs) and *Gr61a* (*Gr61a*+ GRNs). Cell-population-specific overexpression of the wild-type form of *Gr5a* (*Gr5a^+^*) in the *Gr5a* mutant background revealed that *Gr61a*+ GRNs localized on the legs and internal mouthpart critically contribute to food choice but not to meal size decisions, while *Gr5a*+ GRNs, which are broadly expressed in many sugar-responsive cells across the body with an enrichment in the labella, are involved in both food choice and meal size decisions. The legs harbor two classes of *Gr61a* expressing GRNs, one with *Gr5a* expression (*Gr5a+/Gr61a+* GRNs) and the other without *Gr5a*expression (*Gr5a−/Gr61a+* GRNs). We found that blocking the *Gr5a+* class in the entire body reduced the preference for trehalose and blocking the *Gr5a*- class reduced the preference for fructose. These two subsets of GRNsare also different in their central projections: axons of tarsal *Gr5a*+/*Gr61a*+ GRNs terminate exclusively in the ventral nerve cord, while some axons of tarsal *Gr5a*−/*Gr61a*+ GRNs ascend through the cervical connectives to terminate in the subesophageal ganglion. We propose that tarsal *Gr5a+*/*Gr61a+* GRNs and *Gr5a*−/*Gr61a+* GRNs represent functionally distinct sensory pathways that function differently in food preference and meal-size decisions.

## Introduction

Feeding is indeed vital for life unless an organism has an alternative energy source, such as maternally supplied nutrition or host-derived metabolites in the case of parasitism. The adult *Drosophila melanogaster* flies visit plants, particularly rotten fruits, for feeding, as navigated by sensory cues associated with the food (Mansourian et al., 2018). While olfaction and vision are predominant sensory modalities used in exploring foods at a distance (Kim and Dickinson, 2017; Brockmann et al., 2018; Mansourian et al., 2018; Cazalé-Debat et al., 2019) gustation plays a key role in the decision to eat or not to eat (Corfas et al., 2019; Sareen et al., 2021). The gustatory sensory organs are widely distributed along the body, including the mouth part (labella), pharynx, leg tarsal segments, wing margins, and genitalia (Scott, 2018; Montell, 2021). A flying fly will land on a presumptive food source, which likely stimulates gustatory receptors on the leg upon landing. Thus, those receptors expressed in the leg gustatory hairs are primary candidates for sensory channels that contribute to feeding decisions.

The sensory organs express taste receptors belonging to either the gustatory receptor (GR) family of seven-pass transmembrane proteins or the ionotropic glutamate receptor (IR) family (Scott, 2018; Montell, 2021). The *Drosophila melanogaster* genome carries 60GR family genes, and 9GR proteins are known to form a subfamily for sugar taste receptors (Scott, 2018; Montell, 2021). A gustatory sensory hair on the leg tarsus harbors a set of sensory neurons expressing several GRs (abbreviated as GRNs hereafter), the combination of which varies depending on the neuron (Chen and Dahanukar, 2020). However, the exact roles of different receptor proteins and different GRNs on the leg in feeding remain to be explored. A previous study demonstrated that inhibition of *Gr5a*-expressing GRNs had only a modest effect on sugar preference, whereas inhibition of *Gr64f*-expressing GRNs severely impaired the preference (Thoma et al., 2016). These two types of GRNs were also found to send their axons to distinct regions of the central nervous system (CNS; Thoma et al., 2016).

The present article further explores the possible anatomical and functional diversification among GRNs expressing different GRs in feeding behavior; focusing on *Gr5a*-expressing GRNs and *Gr61a*-expressing GRNs, we address whether these two types of GRNs contribute to: (1) the discrimination of sugar species in food choice; and (2) the decision of meal size. Our analysis reveals that blocking*Gr5a*-expressing GRNs disturbs both meal size decisions and food preference, whereas blocking*Gr61a*-expressing GRNs only affects food preference. We confirmed that tarsal *Gr5a*-expressing GRN axons terminate exclusively in the thoracic neuromeres, in contrast to tarsal *Gr61a*-expressing GRNs, some of which extend their axons to the subesophageal zone in the brain. These findings strengthen the view that the primary gustatory afferents mediated by *Gr5a*-expressing GRNs and those mediated by *Gr61a*-expressing GRNs represent separate, yet partially overlapping, sensory channels, each presumably impinging on distinct central circuits that play different roles in food choice and meal size decisions.

## Methods

### Fly Stocks

Flies were reared on a cornmeal medium at 25°C. For the construction of the *Gr61a*-*Gal4* transgene, a ~1 kbp genomic fragment upstream of the *Gr61a*-coding region was PCR-amplified from genomic DNAs extracted from the Canton-S strain using the primers aggatcctgggttgtcctgcctcaaagcac and ctgcggccgctcctcagctctgaccgtcagc for subcloning into pGEM T-easy (Promega). The PCR fragment was introduced upstream of the *Gal4* coding sequence in the pGaTN vector, then introduced into the CaSpeR-4 vector together with the *Gal4* coding sequence for transformation. The *Gr5a* promoter-*Gal4* strain has been described previously (Usui-Aoki et al., 2005). For the construction of the *UAS-Gr5a*^+^ transgene, the whole *Gr5a*-coding region was PCR-amplified and cloned into pGEM-T easy, using template cDNA prepared from the labella of Canton-S and the primers ctgttttattcctcatcactggcc and tacatgccaattagtgcgtct. Then the PCR fragment was introduced into the pUAST vector for transformation. All the transgene constructs were introduced into the *w^1118^* strain by a standard *P*-element-mediated technique (Brand and Perrimon, 1993). The UAS lines carrying *UAS-TNT* or *UAS-IMP-TNT* were kind gifts from Dr. Cahir O’Kane. Female flies were used for the behavioral experiments. Flies collected 3–5 days after eclosion were starved but allowed to take water for 16–18 h before the experiment.

### Histology

Labeling of GRNs expressing *Gr5a* and *Gr61a* was carried out by expressing a variant of the green fluorescent protein mCD8::GFP by means of the *Gal4/UAS* system. Adult flies aged over 10 days after eclosion were used, as these flies had accumulated a sufficient amount of mCD8::GFP. Reporter expression was detected either by direct observation of raw fluorescence under a fluorescence microscope (Microphot-FX; Nikon, Tokyo) or by immunostaining GFP with a rabbit anti-GFP antibody (Molecular Probes, Eugene, OR) in conjunction with a goat anti-rabbit secondary antibody conjugated with Alexa Fluor 488 (Molecular Probes), which was followed by observation under a confocal microscope (BioRad, Hercules, CA). Neuropiles in the CNS were labeled with the mouse monoclonal antibody nc82 (Developmental Studies Hybridoma Bank at the University of Iowa) and a Cy3-conjugated goat anti-mouse secondary antibody (Jackson Immuno Research, West Grove, PA).

### Proboscis Extension Reflex Test

After 18–20 h of food deprivation, flies were affixed to a glass capillary 1 mm in diameter by applying a small amount of wax to the back under cold anesthesia and then left for 2 h in a humid chamber for recovery before the test. Gustatory stimulations were made by touching all the legs simultaneously with a solution droplet provided *via* a micropipette tip. Prior to every sugar stimulation, flies were given a water stimulation with a droplet of water. Flies that extended the proboscis to the water were then allowed to drink until satiated. In a series of tests, 20–24 flies were stimulated one after another by an increasing series of concentrations of trehalose, sucrose, or fructose solutions, and the number of flies that extended the proboscis to a given stimulus was counted. A total of seven to nine tests were performed for each sugar stimulation.

### Two-Choice Feeding Preference Assay

Feeding preference between two different sugar solutions was investigated as described previously (Ueno et al., 2001). Approximately 30 food-deprived flies were given a two-choice test between two sugar solutions, one consisting of 1% agar mixed with a blue food dye (0.125 mg/ml brilliant blue FCF; C_37_H_34_N_2_Na_2_O_9_S_3_; CAS No. 3844-45-9) and the other of 1% agar mixed with a red food dye (0.5 mg/ml acid red 27; C_20_H_11_N_2_Na_3_O_10_S_3_; CAS No. 915-67-3). The flies were offered the choice between solutions for 1 h in the dark. After the test, the flies were frozen and their abdomens were inspected for staining by the ingested dyes. The preference index for sugar solution A (PI_AB_) in the two-choice trial between solution A and solution B was calculated as follows:


$$PI_{\text{AB}}=(N_{\text{A}}+N_{\text{I}}/2)/(N_{\text{A}}+N_{\text{B}}+N_{\text{I}})$$

where N_A_, N_B_, and N_I_ are the number of flies whose abdomens were colored by solution A, solution B, and both solutions, respectively.

### Measurement of Food Intake

Prior to the experiment, the flies were fed with 100 mM sucrose solution for 2 h and then allowed to take only water for 20–22 h for starvation. In the test, approximately 50 food-deprived flies were allowed to consume a solution of sugar and blue dye mixture in 1% agar in the dark for 1 h as in the feeding preference test. After feeding, 20–30 flies were randomly chosen and homogenized in an extraction buffer (75% EtOH/PBS). The dye’s concentration was photometrically determined, and the mean intake per individual fly was then estimated.

### Electrophysiology

Female forelegs were isolated at the tibia and penetrated by a glass capillary filled with a standard saline solution containing 0.75% NaCl, 0.035% KCl, and 0.021% CaCl_2_. The capillary was inserted with a silver wire and served as an indifferent electrode. Another capillary containing sucrose solution dissolved in 0.007% KCl as an electrolyte for conductance was used for sugar stimulations and as a recording electrode. The tarsal sensilla tips were carefully stimulated by contacting the sugar solution to the sensilla tips under a dissection microscope. Extracellular neuronal spike signals recorded from the sensilla tips were amplified by a high impedance (>10^11^ ohm) differential pre-amplifier (MEZ-7200; Nihon Koden, Tokyo). The output was then A/D converted at 100 μs sampling intervals by Power Lab (AD Instruments, Dunedin, New Zealand) for the analysis of the neural activity.

## Results

### Differential Distribution of *Gr5a*-Expressing GRNs and *Gr61a*-Expressing GRNs in the Peripheral Organs

**Figure 1:**
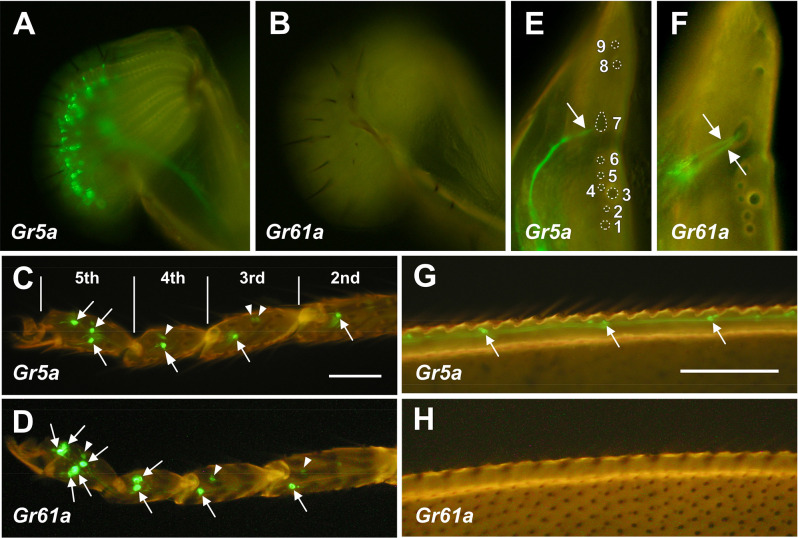
Distributionof*Gr5a*+ and *Gr61a*+ GRNs in peripheral gustatoryorgans. **(A,C,E,G)** Fluorescence microscopic images of*Gr5a+*GRNs of the adult flies with the genotype *w*;*Gr5a*-*Gal4*/*Gr5a*-*Gal4*;*UAS*-*mCD8::GFP*/*UAS*-*mCD8::GFP.Gr5a+* GRNs are widely distributed in gustatory organs: the labial palp **(A)**, tarsal leg segments **(C)**, labral sense organ (LSO) of the pharynx **(E)** and wing margin **(G)**. **(B,D,F,H)** Fluorescence microscopic images of *Gr61a+* GRNs of the adult flies with the genotype *w*;*Gr61a*-*Gal4*/*Gr61a*-*Gal4*;*UAS*-*mCD8::GFP*/*UAS*-*mCD8::GFP*. *Gr61a+* GRNs were localized to the tarsus **(D)** and LSO **(F)**. **(C,D)** Tarsal segments of the prothoracic leg. *Gr5a*+ **(C)** and *Gr61a+*GRNs **(D)** are found in the 2nd, 3rd, 4th, and 5th tarsal segments (arrows). Additionally, small cells also express the reporter (arrowheads). **(E,F)** The LSO. The nine sensilla that make up the LSO are indicated by the numerals 1–9. Dendrites of a single *Gr5a*+ GRN **(E)** and two *Gr61a*+ GRNs **(F)** enter the 7th sensillum of the LSO (arrows). Anterior top and dorsal right view of the images in panels **(E)** and **(F)**.

First, we compared the expression patterns of *Gr5a* and *Gr61a* in GRNs with the aid of respective Gal4 drivers (Brand and Perrimon, 1993). We note that detections of *Gr5a* and *Gr61a* transcripts by *in situ* hybridization were not successful and no antibodies were available for the proteins they encode, as is the case for most other Gr family members. CRISPR/Cas9-mediated knock-in to the *Gr* loci of a reporter would be useful to determine whether these classical Gal4 drivers faithfully recapitulated endogenous expression of the respective proteins. Consistent with previous reports, we found a large number of GRNs expressing *Gr5a-Gal4* (abbreviated as *Gr5a+* GRNs hereafter) in the labella, a major gustatory organ in adult flies (Chyb et al., 2003; Thorne et al., 2004; Wang et al., 2004; Dahanukar et al., 2007; Figure 1A). In contrast, no GRNs expressing *Gr61a-Gal4* (*Gr61a*+ GRNs) were identifiable in the labellar taste sensilla (Figure 1B). However, Dahanukar et al. (2007), Weiss et al. (2011), and Dweck et al. (2021) reported the detection of a few *Gr61a*+ GRNs in the labella, which might have escaped our detection. Comparisons of staining obtained with different *Gr61a-Gal4* lines might help resolve this discrepancy.

In the legs, two pairs of *Gr5a+* GRNs were found in the most distal (5th) segment of the tarsus (Figures 1C and 3D). A single pair of *Gr5a+* GRNs was also present in the 2nd, 3rd, and 4th segments of the foreleg (Figures 1C and 3G). In the midleg and hindleg, a pair of *Gr5a+* GRNswas found in the 4th and 5th segments but no *Gr5a+* GRNs were found in other segments (not shown). To summarize, 10 *Gr5a*+ GRNs were found in the foreleg and four in the other legs. Notably, in the legs, *Gr61a+* GRNs were more abundant than *Gr5a+* GRNs: the 5th tarsal segment of the foreleg carried three pairs of *Gr61a*+ GRNs (Figures 1D and 3E,G), and each of the 2nd, 3rd, and 4th segments carried a single pair of *Gr61a*+ GRNs (Figure 1D). The midleg and hindleg harbored two pairs of *Gr61a*+ GRNs in the 5th segment and 1 pair in the 4th segment (not shown). To summarize, we found a total of 12 *Gr61a*+GRNs in the foreleg, six in the midleg, and six in the hindleg. Although the numbers of *Gr5a*+ and *Gr61a*+GRNs estimated in this study were similar to those reported previously (Chyb et al., 2003; Dahanukar et al., 2007; Weiss et al., 2011; Ling et al., 2014), it should be noted that we excluded from our counts some cells that were smaller than typical GRNs and only weakly fluorescent (e.g., arrowheads in Figures 1C or 1D).

**Figure 2:**
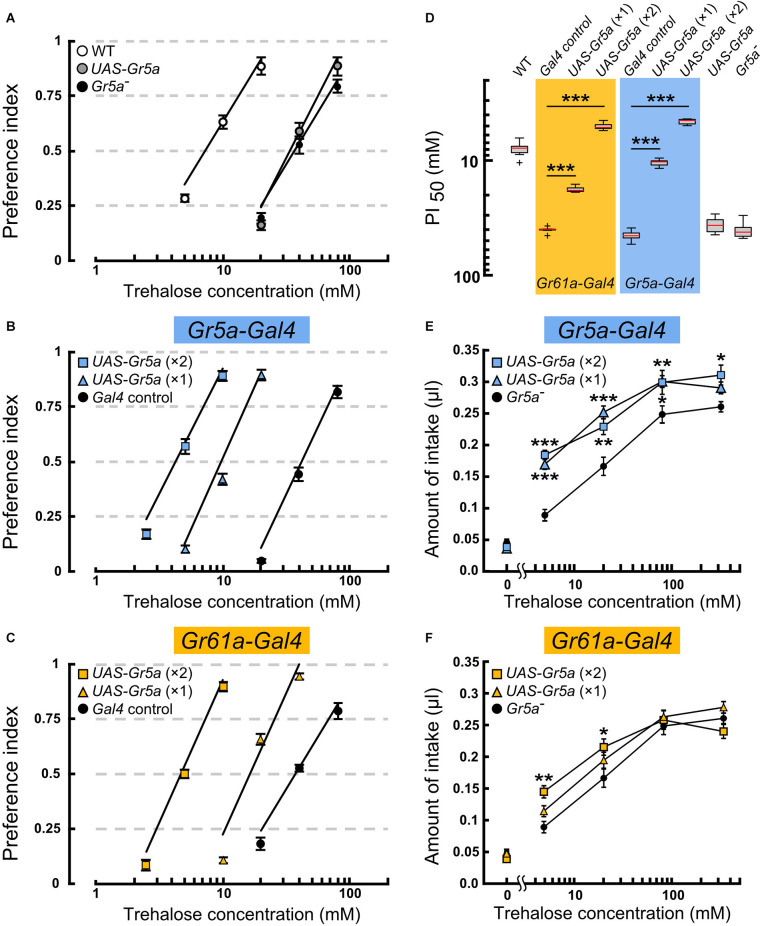
Overexpressionof the *Gr5a^+^* transgene targeted to*Gr5a*+ or *Gr61a*+ GRNs in *Gr5a*^−^ flies differentially modulate ssugar preference and intake. **(A–C)** Feeding preference for various concentrations of trehalose as determined by the two-choice preference test using 2 mM sucrose as a reference. ***p < 0.001 by Welch’s *t*-test with Bonferroni correction (*n* = 7–9 experiments). **(D)** The concentrations of trehalose solution at which trehalose was equally preferred to 2 mM sucrose solution. **(E,F)** Concentration dependence of trehalose intake in flies with *Gr5a^+^* transgene expression as driven by *Gr61a-Gal4* **(E)** or *Gr5a-Gal4* **(F)**. *p < 0.05, **p < 0.01, ***p < 0.001 by Student’s *t*-test (*n* = 7–9 flies).

**Figure 3:**
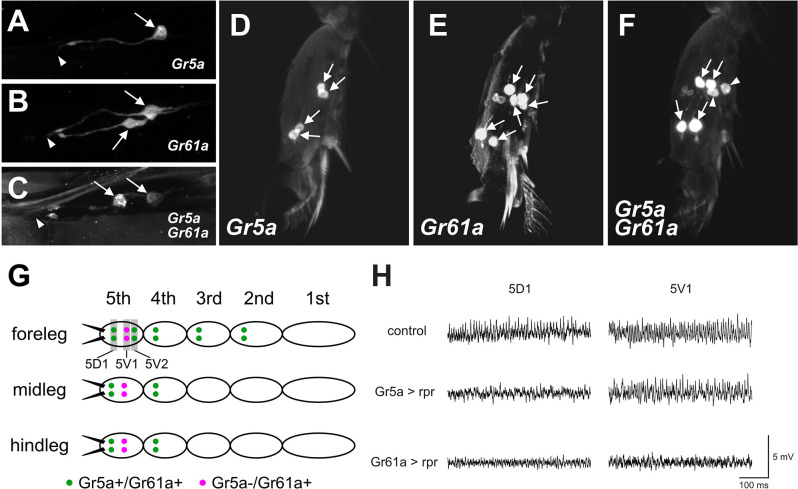
*Gr5a*and *Gr61a* are coexpressed in some GRNs in the LSO and legs.**(A–C)** Projection images of optical sections ofthe LSO. GRNs are labeled by *UAS-mCD8::GFP* driven by *Gr5a-Gal4* **(A)**, *Gr61a-Gal4* **(B)**, and both *Gr5a-Gal4* and *Gr61a-Gal4* **(C)**. Arrows and arrowheads indicate the cell bodies of labeled GRNs and the location of the cuticular pore structure of 7th sensillum of LSO, respectively. **(D–F)** Projection images of optical sections of the 5th tarsal segment of the foreleg. GRNs are labeled by *UAS-mCD8::GFP* driven by *Gr5a-Gal4* **(D)**, *Gr61a-Gal4* **(E)**, and both *Gr5a-Gal4* and *Gr61a-Gal4* **(F)**. **(G)** A schematic illustration of the distribution of *Gr5a*+/*Gr61a*+ GRNs and *Gr5a*−/*Gr61a*+ GRNs in the legs. Ten *Gr5a*+/*Gr61a*+ GRNs are distributed in 2nd to 5th segments of the foreleg, and four *Gr5a*+/*Gr61a*+ GRNs are located in the 4th and 5th segments of the midleg and hindleg (green dots). Two *Gr5a*−/*Gr61a*+ GRNs are distributed in the 5th segment of each leg (purple dots). **(H)** Electrophysiological responses to sugar solution recorded from 5D1 and 5V1 tarsal taste hairs. Representative records obtained from adult females with genotypes *w^1118^*(control), *w^1118^*; *Gr5a-Gal4/UAS-rpr; UAS-mCD8::GFP/+* (Gr5a >rpr) and *w^1118^*; *Gr61a-Gal4/UAS-rpr; UAS-mCD8::GFP/+* (Gr61a >rpr) are shown. 50 mM sucrose solution was used as a stimulant. Female flies were used for all the histological observations and electrophysiological analyses.

In the internal mouthpart, *Gr5a+*GRNs and *Gr61a+*GRNs were found in the labral sense organ (LSO). Among the nine LSO sensilla (Stocker and Schorderet, 1981), the 7th sensillum housed a single *Gr5a*+GRNand two *Gr61a+* GRNs per hemisegment, respectively (Figures 1E,F). *Gr5a+* GRNs were also found in chemosensory neurons on other body parts, including the wing margins (Figure 1G), maxillary palp, and female genitalia (data not shown). No *Gr61a+* GRNs were found in these body parts (Figure 1H).

In summary, *Gr5a+* GRNs and *Gr61a+* GRNs showed distinct distributions across sensory organs. Of note, *Gr5a*+ GRNs were richly distributed in the labella but were less abundant in the leg, whereas *Gr61a*+ GRNs were absent from the labella yet were abundant in the leg.

### *Gr61a*+ GRNs Enhance the Acuity of Feeding Preference but Do Not Promote Intake

To explore the possibility that *Gr5a+*GRNs and *Gr61a+* GRNs make different contributions to feeding, we examined the feeding preference of flies where sensory input to *Gr5a*+ or *Gr61a+* GRNs was selectively enhanced by exogenous expression of a *Gr5a^+^* transgene (Ueno et al., 2001). All preference assays with the transgene were conducted on the *Gr5a^Tre01^* genetic background, which carries a nonsynonymous spontaneous mutation in the coding sequence of *Gr5a* (Ala218Thr: abbreviated as *Gr5a^−^* hereafter), leading to a low gustatory sensitivity to trehalose (Ueno et al., 2001; Inomata et al., 2004). The mutated residue was localized in the deduced second cytoplasmic loop and was found to be responsible for variations in trehalose sensitivity among 152 isomale strains established from natural fly populations. Thus, any host sugar GRNs will potentially be enhanced for the trehalose sensitivity by expressing the *Gr5a*^+^ transgene under *Gal4* control. *Gr5a^Tre01^* represents an allele contributing to natural polymorphism of the *Gr5a* locus, being shared by many *melanogaster* fly stocks including *w^1118^*, a line that often serves as a control in experiments involving *D. melanogaster*. X-chromosomes of *w^1118^* carrying the *Tre^01^*mutation were crossed into the fly stock that had been outcrossed to the CS wild-type strain, resulting in the *Gr5a^Tre01^* line used in this study. However, any potential off-site effect inherent in the use of the *Gr5a^Tre01^* line needs to be evaluated by testing an independent *Gr5a* variant, ideally, one produced by the CRISPR/Cas9 technique. The feeding preference was measured by providing a choice between a trehalose solution of various concentrations and a control 2 mM sucrose solution (Figures 2A–C). The trehalose concentration at which the ratio of the two feeding choices was 50% (PI_50_) was defined as the trehalose sensitivity and compared among flies of different genotypes.

In the host *Gr5a^−^* strain, the PI_50_ was 40.9 ± 5.9 mM (mean ± SEM), while in Canton-S, a wild-type strain carrying a high trehalose sensitivity allele *Gr5a*^+^(*Tre^+^*), it was 8.1 ± 1.3 mM (Figures 2A and 2D). Targeting *UAS-Gr5a^+^* to *Gr5a*+ GRNs by single copies of *Gr5a-Gal4* and* UAS*-*Gr5a^+^* significantly enhanced trehalose preference (Figures 2B,D; PI_50_ = 10.5 ± 0.7 mM). Flies carrying two copies of*Gr5a-Gal4* and two copies of *UAS-Gr5a^+^*showed a trehalose preference even higher than that of the wild type (Figures 2A,B,D; PI_50_ = 4.6 ± 0.3 mM), confirming the contribution of *Gr5a*+ GRNs to the fly’s feeding preference for trehalose. Forced expression of *Gr5a^+^* in *Gr61a*+ GRNs also significantly increased the trehalose preference (Figure 2C). The PI_50_value in flies with one copy and that in flies with two copies of each of *Gr61a-Gal4* and *UAS*-*Gr5a^+^* were 17.8 ± 0.9 mM and 5.1 mM ± 0.3 mM, respectively (Figure 2D). The latter value again shows a significantly higher trehalose preference in these flies than in the wild type. Thus, depending on the gene dosage, the *UAS-Gr5a^+^*transgene enhanced the trehalose preference when selectively expressed in *Gr61a+*GRNs, demonstrating that *Gr61a*+ GRNs made a substantial contribution to the feeding preference. An obvious caveat is that *Gr5a^+^* exogenously expressed in *Gr5a*- GRNs might affect the functioning of endogenously expressed Grs, and thereby result in an enhanced trehalose preference, although it has been reported that two different sugar-responsive Grs present in single GRNs operate independently of one another (Dahanukar et al., 2007).

Using the same transgenes, we next examined whether *Gr5a+* GRNs and *Gr61a+* GRNs are involved in the regulation of food intake by quantitatively measuring the amounts of ingested trehalose solutions. A *Gr5a*^−^ fly consumed, on average, 0.046 ± 0.005 μl sugar-free water during a 1 h test period, and the amount of intake increased when trehalose was added at increasing concentrations in the range of 5–80 mM (Figure 2F). Transgenic expression of *Gr5a^+^* in *Gr5a*+ GRNs with single copies of *Gr5a-Gal4* and *UAS-Gr5a^+^*significantly increased the amount of intake of trehalose solutions at concentrations of 5, 20, and 80 mM (Figure 2E). Flies with two copies of *Gr5a-Gal4* and *UAS-Gr5a^+^* showed an increase in the amount of intake even at the trehalose concentration of 320 mM. Therefore, enhanced trehalose sensitivity in *Gr5a*+ GRNs is sufficient to stimulate the consumption of trehalose solutions with a wide range of concentrations. Next, we examined whether heterologous expression of the *Gr5a^+^* transgene in *Gr61a*+ GRNs similarly promotes trehalose intake. In contrast to the case of transgenic expression in *Gr5a+* GRNs, transgenic expression in *Gr61a*+ GRNs did not increase the consumption of trehalose solutions at any concentration tested in flies with single copies of *Gr61a-Gal4* and *UAS*-*Gr5a^+^* (Figure 2F). In the flies with two copies of *Gr61a-Gal4* and *UAS*-*Gr5a^+^*, a significant increase in the intake was observed only at 5 and 20 mM (Figure 2F). These observations indicate that *Gr61a+* GRNs play a limited role in the control of meal size. We conclude that, whereas *Gr5a*+ GRNs make a significant contribution to both food choice and meal size control, *Gr61a*+ GRNs are more important for food choice with only a marginal effect on the determination of meal size.

### *Gr5a* and *Gr61a* Define Two Anatomical Classes of GRNs

The functional differences found between *Gr5a* + GRNs and *Gr61a* + GRNs in meal size control and food choice may suggest that at least two distinct GRN populations are differently involved in these two behavioral decisions. To obtain clues to the cellular identity of such GRN populations, we employed double-labeling of GRNs with *Gr5a-Gal4* and *Gr61a-Gal4* in the LSO and the legs, and counted the number of GRNs that express the respective GAL4 (Figure 3).

When the LSO was singly stained for *Gr5a*, only one cell per hemisegment in the 7th sensillum was labeled, in contrast to the LSO stained for *Gr61a*, in which two cells were labeled (Figures 3A,B). In flies carrying both *Gr5a-Gal4* and *Gr61a-Gal4*, we found two GRNs that were positive for Gal4 (Figure 3C). One of the two neurons was more intensively labeled than the other, suggesting a higher level of *Gal4* expression in the neuron. We inferred that the intensively labeled GRNwas double-positive for *Gr5a* and *Gr61a* (*Gr5a*+/*Gr61a*+), while the less intensively labeled GRN expressed only *Gr61a-Gal4* (*Gr5a*−/*Gr61a*+).

Similar analyses in the 5th tarsal segment of the foreleg (Figures 3D–F) suggested that, among the three pairs of GRNs that were positive for *Gr61a*, two pairs also expressed *Gr5a* (*Gr5a*+/*Gr61a+*), while a single pair of GRNs expressed only *Gr61a* (*Gr5a*−/*Gr61a+*): more specifically, one of the two proximal neuron pairs (Figure 3F, arrowheads) was singly positive for *Gr61a* (*Gr5a*−/*Gr61a*+), whereas the other proximal pair and one distal pair were positive for both *Gr5a* and *Gr61a* (*Gr5a*+/*Gr61a*+: Figure 3F, arrows) in the 5th tarsal segment. In the 5th tarsal segment of the mid- and hindleg, one proximal pair was positive only for *Gr61a* (*Gr5a*−/*Gr61a*+) and one distal pair was positive for both *Gr5a* and *Gr61a* (*Gr5a*+/*Gr61a*+). It was inferred that GRNs on the 2nd and 3rd tarsal segments of the foreleg and those on the 4th tarsal segment of the midleg and the hindleg were *Gr5a*+/*Gr61a*+, since the numbers of labeled GRNs were not increased by double labeling with *Gr5a*-*Gal4* and *Gr61a-Gal4* (not shown). The overall expression profiles of *Gr5a*-*Gal4* and *Gr61a-Gal4* in the leg GRNs are schematically illustrated in Figure 3G.

Previous studies have revealed that four pairs of gustatory hairs are located on the 5th tarsal segment (Meunier et al., 2003; Miyamoto and Amrein, 2013; Miyamoto et al., 2013), and three of them express*Gr61a+* GRNs and innervate the hairs (Miyamoto and Amrein, 2013). We confirmed that two sets of paired *Gr5a*+/*Gr61a*+ GRNs innervated both the 5D1 and 5V2 sensilla, and one pair of *Gr5a*−/*Gr61a*+GRNs innervated the 5V1 sensillum (Miyamoto and Amrein, 2013). To further demonstrate that the 5V1 and 5D1 sensilla contain both *Gr5a−/Gr61a+* GRNs and *Gr5a+/Gr61a+* GRNs, we carried out an electrophysiological analysis. In wild-type flies, 50 mM sucrose elicited impulse discharges in the 5D1 and 5V1 sensilla (Figure 3H; upper row). Expression of the apoptosis gene *reaper* (*rpr*) as driven by *Gr5a-Gal4* selectively abolished the sucrose response in 5D1, indicating that the sugar GRN innervating 5D1 was *Gr5a*-positive, while the sugar GRN innervating 5V1 was *Gr5a*-negative (Figure 3H; middle row). In contrast, expression of *rpr*as driven by *Gr61a-GAL4* abolished the sucrose response in both 5D1 and 5V1, indicating that both 5D1 and 5V1 were innervated by sugar GRNs positive for *Gr61a* (Figure 3H; bottom row). Based on these observations, we conclude that a single, sugar-sensitive cell of the sensillum 5V1 belongs to the *Gr5a*−/*Gr61a*+ GRNs, whereas 5D1 contains *Gr5a*+/*Gr61a*+ GRNs. We postulate that the *Gr5a*+/*Gr61a*+ GRNs associated with 5D1 impact both the meal size and food preference, whereas the *Gr5a*−/*Gr61a*+ GRN associated with 5V1 impact only food preference.

### *Gr5a*+ GRNs and *Gr61a*+ GRNs Display Different Sugar Specificity

**Figure 4:**
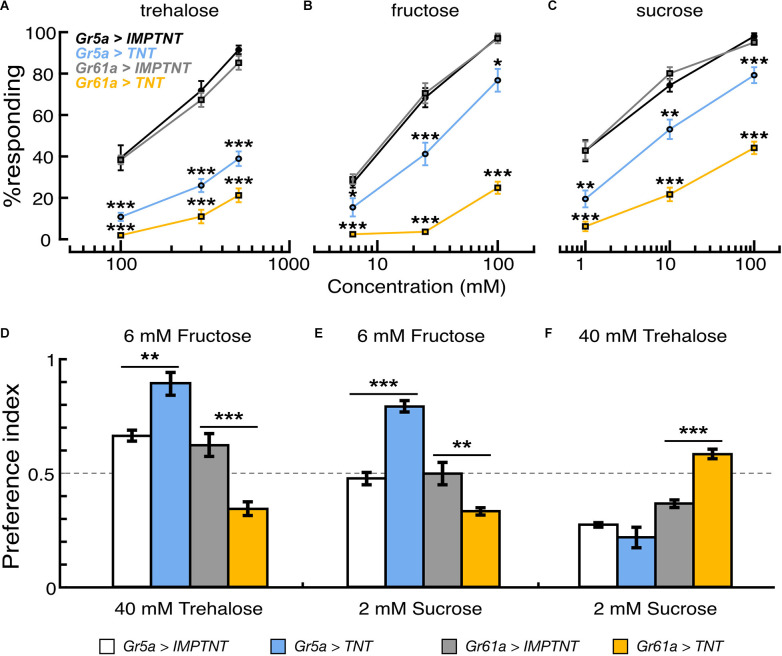
Effectof population-specific blocking of sugar GRNs on feeding reflex and sugar preference. **(A–C)** Proportion of flies that showed proboscis extension reflex to tarsal stimulation with trehalose, fructose, and sucrose solutions in flies where synaptic transmission of *Gr5a+* or *Gr61a+*GRNs are blocked by ectopic expression of TNT. PER responses are shown as the average for those flies that responded in seven to nine experiments. In each experiment, 20–25 flies were used. **(D–F)** Feeding preferences in response to different combinations of sugar solutions. In each panel, scores are indicated as the average feeding preference for the solutions indicated on the top of the panel. Error bars indicate SEM. *p < 0.05; **p < 0.01; ***p < 0.001 by *U*-test.

To address whether *Gr5a*−/*Gr61a*+GRNs and *Gr5a*+/*Gr61a*+ GRNs differently contribute to sugar detection in the legs, we performed proboscis extension reflex (PER) assays with sugar stimulations onto the legs in the flies where the synaptic transmission of *Gr5a*+ GRNs or *Gr61a*+ GRNs was blocked by targeted expression of tetanus toxin (Sweeney et al., 1995). The stimulus–response relations for three sugar species are shown in Figures 4A–C. The effective concentrations to elicit the PER response in control flies differed among the sugar species. The stimulus–response relations in flies with overexpression of the inactive toxin (IMPTNT) in *Gr5a*+ GRNs were indistinguishable from those in flies with IMPTNT overexpression in *Gr61a*+ GRNs (*p* > 0.05; *t*-test). Block of *Gr5a*+ GRNs significantly reduced the PER responses to all three sugars. The reduction was most striking for the response to trehalose solutions: for example, the proportion (%) of responding flies decreased from 91.5% ± 2.1% to 38.9% ± 3.5% at the trehalose concentration of 500 mM upon block of *Gr5a*+ GRNs (Figure 4A). In contrast, the effects of blocking *Gr5a*+ GRNs on the PER responses to fructose or sucrose solutions were weaker, although there were still significant reductions in the response under all conditions examined (Figures 4B,C). At the maximum concentration examined, the proportion of flies responding was reduced from 97.5% ± 0.9% to 76.8% ± 5.5% when fructose was used as a stimulant, while the proportion was reduced from 98.0% ± 1.5% to 79.2% ± 3.8% when sucrose was used. We note that, in the flies with the same genotype, PER responses to labellar sugar stimulation were abolished (Supplementary Figure 1), and therefore, *Gr5a-Gal4* drove TNT expression at a level sufficient to block synaptic output from *Gr5a*+ GRNs. This means that the leg PER responses retained in flies with TNT expression must be mediated by GRNs that lack *Gr5a* expression (i.e., Gr5a-GRNs). In keeping with this idea, PER responses to tarsal sugar stimulation were more severely disrupted when TNT expression was driven by *Gr61a*-*Gal4* than when TNT expression was driven by *Gr5a-Gal4* (Figures 4A–C), presumably because a larger number of GRNs, including *Gr5a*−/*Gr61a*+ GRNs, were inactivated. In this case, virtually no PER was observed when any of the sugars were used at the lowest concentrations, and the proportion of responding flies was 21.2% ± 3.3%, 24.9% ± 2.9%, and 44.1% ± 3.0% for trehalose, fructose, and sucrose, respectively, even at the maximum stimulant concentrations. These results demonstrate that *Gr5a*−/*Gr61a*+ GRNs, as well as *Gr5a*+/*Gr61a*+ GRNs, mediate the detection of all three sugar species in the legs for feeding responses.

Reductions in PER responses by TNT expression were evaluated by the ratio a/b (where “a” is the PER score for the test-group flies expressing TNT and “b” is the PER score for the control-group flies expressing IMPTNT). In *Gr61a*+ GRNs, the reduction in PER responses was larger when the stimulant was trehalose (a/b was 0.25 at the highest sugar concentration tested) or fructose (0.26) than when it was sucrose (0.46), whereas, in *Gr5a*+ GRNs, the reduction in PER responses was largest with trehalose (0.43), followed by fructose (0.79) and sucrose (0.81; Figures 4A–C). These results suggest that *Gr5a*−/*Gr61a*+ and *Gr5a*+/*Gr61a*+ GRNs mediate PER with different response spectra, i.e., *Gr5a*+/*Gr61a*+ GRNs are better tuned to trehalose while *Gr5a*−/*Gr61a*+ GRNs are better tuned to fructose, and to a lesser extent, to sucrose. Changes in the relative effectiveness of the three sugars in PER induction upon TNT expression presumably reflect wiring differences between *Gr5a-* and *Gr5a+* GRNs in the central nervous system, because TNT likely blocked transmitter release from the synaptic terminals of GRNs with little effect on Gr functions in peripheral dendrites.

We next examined the effect of blocking these GRNs on feeding preference. The same transformants as used in the PER experiment were subjected to the feeding preference assays using 40 mM trehalose, 6 mM fructose, and 2 mM sucrose solutions. Interestingly, the flysugar preference differed according to whether either*Gr5a+* GRNs or *Gr61a+* GRNs were blocked. When given a choice between 6 mM fructose and 40 mM trehalose, flies preferred fructose over trehalose when *Gr5a+* GRNs were blocked, but they preferred trehalose over fructose when *Gr61a+* GRNs were blocked (Figure 4D). Similarly, blocking *Gr5a+* GRNs shifted the feeding preference towards 6 mM fructose against 2 mM sucrose, whereas blocking *Gr61a+* GRNs shifted the preference towards 2 mM sucrose (Figure 4E). When the flies were given a choice between 40 mM trehalose and 2 mM sucrose, inactivating *Gr61a*+ GRNs shifted the feeding preference towards 40 mM trehalose against 2 mM sucrose. Under these choice conditions, blocking *Gr5a+* GRNs did not change the preference significantly (Figure 4F). These results indicate that *Gr61a+* GRNs substantially contribute to the feeding preference and are tuned differently from *Gr5a*+ GRNs to enhance feeding preference for specific sugars (i.e., fructose and, to a lesser extent, sucrose). The response profiles of the tarsal sugar GRNs obtained in the PER experiment are consistent with the fly’s feeding preference, suggesting that the gustatory sensilla on the legs play an important role in food choice.

### Tarsal *Gr5a*−/*Gr61a*+ and *Gr5a*+/*Gr61a*+ GRNs Have Discrete Central Projection Targets

The above experiments revealed that *Gr5a*−/*Gr61a*+ GRNs and *Gr5a*+/*Gr61a*+ GRNs in the legs have different yet overlapping behavioral roles and sugar sensitivity. To pursue the possibility that these differences stem from different networks formed by the two GRN groups, we compared the projection patterns of *Gr5a*+ GRNs and *Gr61a*+ GRNs in the CNS.

**Figure 5:**
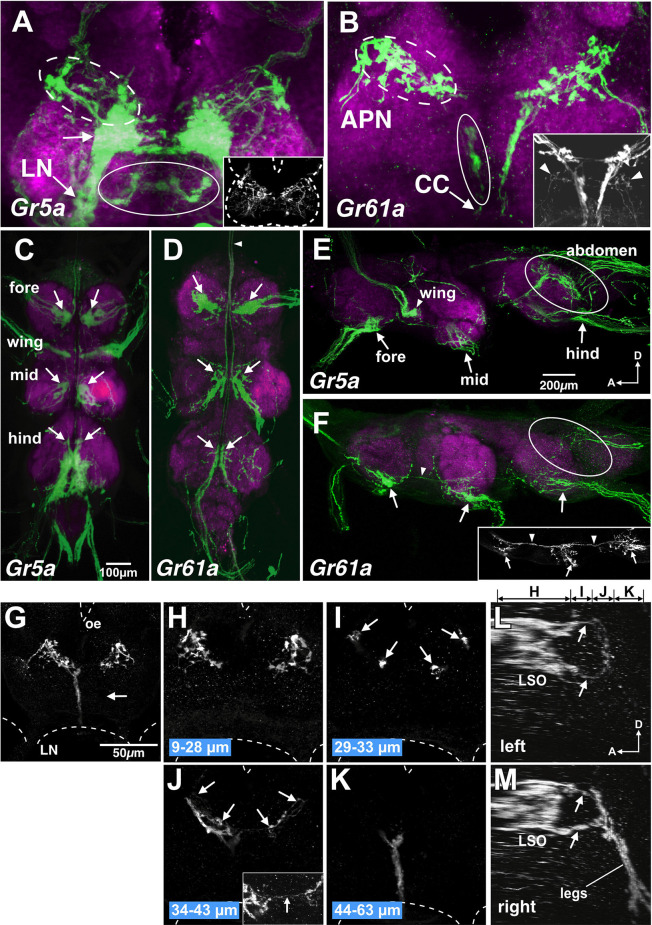
*Gr5a+* GRN axons project exclusively to thoracic neuromeres and subsets of *Gr61a*+ GRNs project to the brain. **(A,B)** SOG doubly stained with anti-GFP (green) and nc82 monoclonal antibodies (magenta) from flies with genotypes of *w*; *Gr5a*-*Gal4*; *UAS-mCD8::GFP* **(A)** and *w*; *Gr61a*-*Gal4*; *UAS-mCD8::GFP* **(B)**. **(A)** A large stained cluster in the medial region (arrow) represents projections of axons of *Gr5a*+ GRNs from labella. In addition, *Gr5a+* afferents *via* the labial nerve (LN) are found in dorsolateral (dotted ellipse), ventral and posterior (ellipse) and anterior (inset) regions in the SOG. Dotted lines in the inset indicate the approximate outline of the brain. **(B)** *Gr61a*+ GRNs entering the brain *via* the accessory pharyngeal nerve (APN) mainly project to the anterior SOG (dotted ellipse), and those entering *via* the cervical connective (CC) mainly project to the posterior (ellipse) SOG. **(C–F)** The ventral nerve cord (VNC) stained with the anti-GFP antibody (green) and the nc82 monoclonal antibody (magenta). The fly genotypes are: *w*; *Gr5a*-*Gal4*; *UAS-mCD8::GFP* **(C,E)** and *w*; *Gr61a*-*Gal4*; *UAS-mCD8::GFP* **(D,F)**. Peripheral origins ofcentral projection clusters are indicated for GRNs in the foreleg,midleg, and hindleg. Arrows indicate the projection of tarsal *Gr5a*+GRNs and *Gr61a*+ GRNs. **(C,D)** Ventral view of the VNC. Anterior is on top. The arrowhead in **(D)** shows the axon bundles of *Gr61a* GRNs that project to the brain. **(E,F)** Sagittal view of the VNC. Anterior is on the left and dorsal on top. Ellipses show the projection of GRNs in the abdomen. Arrowheads in **(F)** indicate the axon bundles of *Gr61a+* GRNs that run anteriorly to reach the brain. **(G–M)** The SOG stained with the anti-GFP antibody. The tissue was taken from flies with the genotype *w*; *Gr61a*-*Gal4*; *UAS-mCD8::GFP*, which were aged at least 10 days after the amputation of left tarsal segments. Frontal view of the SOG zone of the brain. Dotted lines show the approximate outline of the brain. Oe, esophagus; LN, labial nerve. **(G)** A collapsed image of whole serial optical sections. Projections from the legs are absent on the left side (arrow). **(H–K)** Collapsed images of substacks at different depths along the anterior-posterior axis. The distances from the anterior border of the brain are indicated below the images. Arrowheads in **(I)** and **(J)** indicate the axons of *Gr61a*+ GRNs in the LSO that terminate in close proximity to the axon terminals of tarsal *Gr5a*−/*Gr61a* GRNs. The inset in **(J)** shows the region near the midline at a higher magnification. Arrows in the inset show the axons of *Gr61a+* GRNs in the LSO that cross the midline. **(L,M)**. Sagittal view of the SOG reconstructed from same serial images as **(G–K)**. Panels **(L)** and **(M)** show the left and right side, respectively. Above panel **(L)**, the ranges along the anterior–posterior axis covered by the images shown in **(H–K)** are indicated.

Consistent with the previous reports, *Gr5a+* GRNs exhibited somatotopic projections (Wang et al., 2004; Dahanukar et al., 2007; Kwon et al., 2014): the axon terminals of *Gr5a+* GRNs in the foreleg, midleg and hindleg were confined in the ventral region of prothoracic, mesothoracic, and metathoracic neuromeres in the ventral nerve cord (VNC; arrows in Figures 5C,E), respectively, and *Gr5a+* GRNs in the labella and LSO were in the subesophageal ganglion (SOG; Figure 5A; Scott et al., 2001; Wang et al., 2004; Dahanukar et al., 2007; Miyazaki and Ito, 2010). Likewise, *Gr5a+* GRNs on the wing margin projected to the accessory mesothoracic neuromere (Figure 5C). In addition, the dorsal portion of the abdominal neuromere harbored neurites of *Gr5a*+ GRNs, which likely innervate taste hairs in the genitalia (circle in Figure 5E).

As in the case of *Gr5a*+ GRNs, some *Gr61a+* GRNs project to ventral neuromeres in the VNC (arrows in Figures 5D,F), with fewer axons that terminate in the dorsal part of the metathoracic neuromere (ellipses in Figure 5F). In contrast to *Gr5a*+ GRNs, however, bilateral axon bundles of *Gr61a*+ GRNs were clearly observed along the midline, running through connectives between segments and the cervical connective, as described previously (Dahanukar et al., 2007). The bundle size within a thoracic neuromere appeared larger in progressively anterior ganglia and was largest in the cervical connective (arrowhead in Figure 5F), suggesting that *Gr61a+* GRN afferent fibers from each leg fasciculate with each other to form a bundle and project to the brain together. Since all *Gr5a*+ GRNs in each leg form terminals within the respective thoracic neuromere without extending ascending axons to the brain, *Gr61a+* GRN axons terminating in the VNC must be those of the *Gr5a*+/*Gr61a*+ GRN population. Conversely, all *Gr61a+* axons that ascend to the brain represent those of *Gr5a−/Gr61a+* GRNs.

In the SOG, in contrast to*Gr5a+* GRNs, *Gr61a+* GRNs make only a marginal contribution to the labial nerve bundle (arrowheads in the inset in Figure 5B), which is in line with the observation that few *Gr61a+* GRNs were found in the labella (Figure 1B). Instead, the axons of leg *Gr5a*−/*Gr61a*+GRNs form a thick bundle running across the ventral posterior and dorsal anterior SOG (Figure 5B). Afferents of *Gr61a+* GRNs were also observed in the accessory pharyngeal nerve that enters the anterior SOG (Figure 5B, dotted circle), and these fibers likely originate from *Gr61a+* GRNs in the LSO (Figure 1F). We were unable to rigorously identify the afferents of *Gr5a+* GRNs from the LSO, due to the abundant labellar *Gr5a*+ GRNs that hampered the detection of the former (Figure 5A, dotted circle).

Notably, axons of tarsal *Gr5a*−/*Gr61a*+ GRNs terminated close to the region where LSO *Gr61a+* GRN axons terminated. To discriminate terminals of the two populations, we ablated all the left tarsal segments of a fly in which *Gr61a*+ GRNs were labeled. We found that only the left posterior and ventral projection areas were eliminated, while the right side of the SOG remained intact (Figures 5G–K). The right and left projection areas of the anterior and dorsal SOG were also kept intact (Figure 5H). We conclude that the posterior projection originates from *Gr5a*−/*Gr61a+* GRNs in the ipsilateral tarsus and that the anterior dorsal projection originates from the *Gr61a+* GRNs in the LSO. The most anterior axon termini of the tarsal *Gr5a*−/*Gr61a+* GRNs and the most posterior axon termini of the *Gr61a+* GRNs in the LSO were both bifurcated (Figures 5L,M), terminating on two target regions so that fibers from the leg and LSO were juxtaposed with each other (arrows in Figure 5M). Therefore, inputs from *Gr5a*−/*Gr61a*+ GRNs in the legs and *Gr61a*+GRNs in the LSO converge onto the same region of the brain despite originating from distinct peripheral locations. We conclude that *Gr5a−/Gr61a*+ GRNs and *Gr5a+/Gr61a* GRNs form different central connections to convey sensory codes for sugars to separate neural pathways that operate under distinct behavioral contexts.

## Discussion

Our behavioral test in flies expressing the*Gr5a^+^* transgene in *Gr5a*+ or *Gr61a*+ GRNs revealed that *Gr61a*+ GRNs play a major role in feeding preference but make only a small contribution to intake control (Figures 2C and 2E), demonstrating their specialized role in food choice. The taste hairs exist throughout the body surface, including the labella, legs, wing margin, and female genitalia. Among these, taste hairs on the legs are most suited to detecting substances on the ground. *Gr5a*−/*Gr61a*+ GRNs are richly distributed on leg tarsi, and thus likely mediate the detection and evaluation of sugars. *Gr5a+/Gr61a-*GRNs, in contrast, are scarce on the legs, yet *Gr5a^+^* overexpression in *Gr5a*+ GRNs enhanced, as demonstrated in this article, both the preference for and intake of trehalose. *Gr5a*+/*Gr61a−* GRNs are enriched in the labella, making them promising candidates for the GRNs responsible for these enhancing effects. Touching food with a leg triggers a PER response that stimulates a second contact with food but *via* the labellum, leading to a recursive induction of PER responses. It appears that interactions between the leg *Gr5a*−/*Gr61a*+ and the labellar *Gr5a*+/*Gr61a*− GRNs through such a feedback loop may sustain feeding, and thereby increase food intake.

Our results unraveled the logic underlying the organization of gustatory projections in the central nervous system (CNS) of the fly: we hypothesize that gustatory projections segregate or converge in the CNS not because the GRNs encode different taste categories or because GRNs are localized to different bodily sites. Rather, projections segregate or converge based on the behavioral processes to be regulated by the given gustatory inputs. This is why tarsal *Gr5a*−/*Gr61a*+ GRNs and *Gr5a*+/*Gr61a*+ GRNs terminate in different CNS regions, i.e., the VNC and SOG, respectively, even though both GRN groups innervate the tarsal sensilla (Figures 5B,D). Conversely, tarsal *Gr5a*−/*Gr61a*+ GRNs and *Gr61a*+ GRNs in the LSO converge on to a SOG site, although they are located in different appendages (Figures 5L,M). These patterns of gustatory projections are likely correlated with distinct contexts, in which given gustatory inputs are required for regulating a fly behavior. In fact, PER was not the sole response when the tarsal *Gr5a*+/*Gr61a*+ GRNs were stimulated by sugar. The same stimulus may induce an orientation response to the food source as guided by gustatory inputs originated from sensilla on a particular leg, which drives a motor circuit for locomotion within the VNC. In fact, Thoma et al. (2016) proposed that GRNs projecting only to the VNC mediate sugar-mediated suppression of locomotion. It is an interesting possibility that such reduced motility increases the dwell time on food, resulting in an increase in meal size. Tarsal *Gr5a*−/*Gr61a*+ GRNs, on the other hand, could be involved in food evaluation when their ascending axons convey inputs to the SOG site, where inputs from LSO *Gr61a*+ GRNs also impinge; in theory, spatial information would not be retained there if two inputs with spatially distinct origins were overlaid. These considerations suggest that *Gr5a*−/*Gr61a*+ GRNs and *Gr5a*+/*Gr61a*+ GRNs respectively contribute to two different pathways, one dedicated to the regulation of meal size decisions, and the other participating in the selection of specific foods. *Ir60b*-expressing sugar-sensitive GRNs and *Gr43a*-expressing sugar-sensitive GRNs in the pharynx inhibit IN1 interneurons in the SOG to stop feeding (Yapici et al., 2016; Yang et al., 2021), whereas a pair of dopaminergic neurons in the SOG modulate the motivation to consume sucrose (Marella et al., 2012). It remains to be examined whether the two sensory pathways that convey sugar codes originating from the labella and legs, and which were reported herein, are wired together with these central neurons involved in the feeding regulation.

## Data Availability Statement

The datasets generated for this article are available from the corresponding authors upon reasonable request.

## Author Contributions

All the experiments were performed by SK except for the electrophysiological experiment, which was performed by NT. SK and KI contributed to the conception of the work, the interpretation of the results and the manuscript preparation. DY reviewed and edited the draft. All authors contributed to the article and approved the submitted version.

## Conflict of Interest

The authors declare that the research was conducted in the absence of any commercial or financial relationships that could be construed as a potential conflict of interest.

## Publisher’s Note

All claims expressed in this article are solely those of the authors and do not necessarily represent those of their affiliated organizations, or those of the publisher, the editors and the reviewers. Any product that may be evaluated in this article, or claim that may be made by its manufacturer, is not guaranteed or endorsed by the publisher.
