# Supplementary material for: Which Sugar to Take and How Much to Take? Two Distinct Decisions Mediated by Separate Sensory Channels
Source: Front Mol Neurosci. 2022 Jun 3;15:895395. doi: 10.3389/fnmol.2022.895395 (PMC9206540; doi:10.3389/fnmol.2022.895395)
Supplement: Supplementary file 1 [file Image_1.pdf]

Supplementary information

Which sugar to take and how much to take? Two distinct decisions mediated by separate sensory channels

Soh Kohatsu, Noriko Tanabe, Daisuke Yamamoto and Kunio Isono

This pdf file includes:

Supplementary Figure 1 and its legend.

Supplementary figure 1

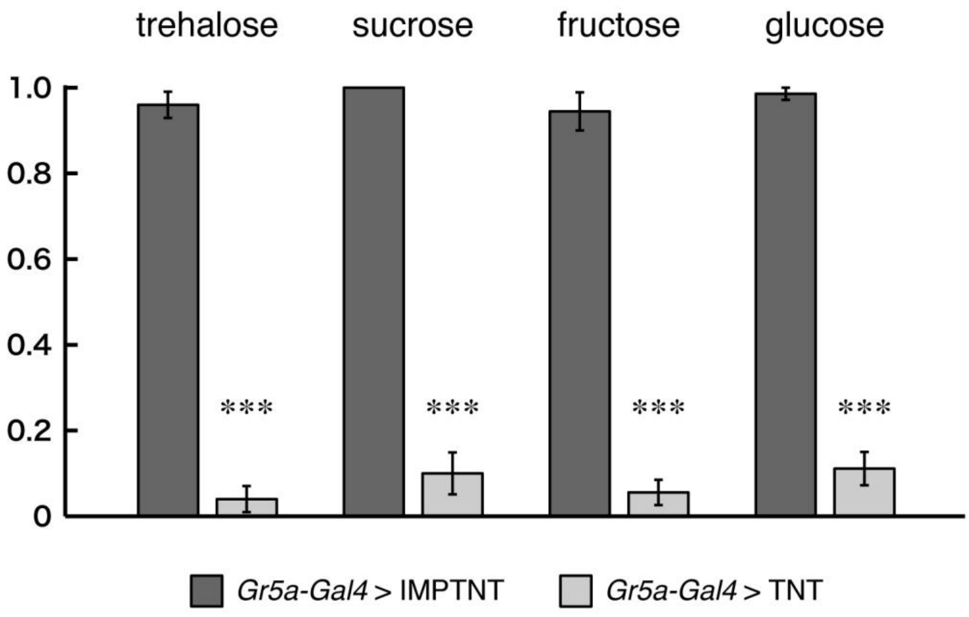

Supplementary Figure 1 Targeted expression of TNT in *Gr5a+* GRNs abolishes PER to labellar sugar stimulation. Proportions of flies that showed proboscis extension reflex to labellar sugar stimulation in flies where synaptic transmission of *Gr5a+* or *Gr61a+* GRNs are blocked by ectopic expression of TNT. Sugars used as stimulant are indicated on the top. Concentration of all sugar solutions was 300 mM. PER response are indicated as average of proportion of flies that showed response in 7 to 10 experiments. Error bars indicate SEM. In each experiment, 20 to 25 flies are used. ***p<0.001; U-test.
